# Additive integer-valued data envelopment analysis with missing data: A multi-criteria evaluation approach

**DOI:** 10.1371/journal.pone.0234247

**Published:** 2020-06-11

**Authors:** Chunhua Chen, Jianwei Ren, Lijun Tang, Haohua Liu

**Affiliations:** 1 School of Business Administration, Jiangxi University of Finance and Economics, Nanchang, Jiangxi, China; 2 Inner Mongolia Branch of Agricultural Bank of China, Hohhot, Inner Mongolia, China; 3 Transportation Institute, Inner Mongolia University, Hohhot, Inner Mongolia, China; 4 School of Mathematical Sciences, Inner Mongolia University, Hohhot, Inner Mongolia, China; 5 School of Business, University of Plymouth, Plymouth, Devon, United Kingdom; Shandong University of Science and Technology, CHINA

## Abstract

Traditional data envelopment analysis (DEA) models assume that all the inputs and outputs data are available. However, missing data is a common problem in data analysis. Although several scholars have developed techniques to conduct DEA with missing data, these techniques have some disadvantages. A multi-criteria evaluation approach is proposed to measure the efficiency of decision making units (DMUs) with missing data. In this approach, analysts first estimate the upper and lower bounds of DMUs’ efficiency using the proposed I-addIDEA-U models (interval additive integer-valued DEA models with undesirable outputs) that can be applied to address integer-valued variables and undesirable outputs. Then, DMUs’ “relative” efficiency is evaluated using the proposed “Halo + Hot deck” DEA method (if there is no correlation between variables) or regression DEA techniques (if there is a correlation between variables). Finally, the multi-index comprehensive evaluation method is applied to determine which scenario (the lower bound of efficiency, the “relative” efficiency, or the upper bound of efficiency) should be selected. With a case study, it is shown that the proposed multi-criteria evaluation approach is more effective than traditional approaches such as the mean imputation DEA method, the deletion DEA method, and the dummy entries DEA method.

## 1. Introduction

Traditional data envelopment analysis (DEA) models assume that all the inputs and outputs data are available [1, 2]. If the data related to some vital variables of decision making units (DMUs) are missing, traditional DEA models cannot be applied to measure the performance of these DMUs [3, 4]. However, missing data is a common problem in data analysis [5].

To deal with the problem of missing data many methods have been proposed, e.g., deletion, imputation, and multiple imputation [6, 7]. (1) The deletion methods (deleting all variables with missing data or all units with missing data) are easy to implement, but they may lead to biased estimates [8]. (2) The imputation methods mainly include the mean imputation, Hot deck imputation, and regression imputation [9, 10]. Mean imputation means that the missing data are replaced by the mean of the available data. It is simple, but the variability in the dataset is reduced [11]. In the Hot deck imputation method, missing data are replaced with the available values from a “similar” unit. Hot deck imputation is an effective method and has been widely used in practice [12, 13]. Regression imputation is also a widely used method in which missing data are replaced with the values obtained from regression techniques, e.g., linear regression, logistic regression, polynomial regression, Probit regression, and Tobit regression [14, 15]. (3) Multiple imputation is also an attractive method, which has been regarded as a more accurate and less biased method [16]. According to the multiple imputation method, missing data should be imputed based on the distributions and variability of other data elements in the sample [17]. (4) There are also some other methods for dealing with missing data, e.g., the maximum likelihood [18, 19], Bayesian [20, 21], and the expectation maximization [22, 23].

Several scholars have researched DEA with missing data in different ways. O’neal et al. applied the deletion method and proposed DEA models (the deletion DEA) to measure DEA efficiency, but this approach was problematic because deleting DMUs may lead to changes in the other DMUs’ relative efficiency [24]. Kuosmanen used dummy entries (zero for output variables and large enough numbers for input variables) to reduce the effects of DMUs with missing data on the relative efficiency of the other DMUs [25]. Gardijan and Lukač applied the dummy entries method and proposed DEA models (the dummy entries DEA) to measure the efficiency of the food and drink industry [26]. Interval DEA approach is another widely used method in which missing data are replaced with a lower bound and an upper bound so that the lower and upper bounds of efficiency can be evaluated [27–29]. Kao and Liu developed a fuzzy DEA approach that allowed analysts to use the available data to evaluate membership functions of fuzzy efficiency [30]. In fact, the fuzzy DEA approach is similar to the interval DEA approach. The difference between the two approaches is that the fuzzy DEA approach is based on the fuzzy theory while the interval DEA approach uses deterministic techniques [31–36]. Zha et al. developed a Halo DEA approach (Halo effect is a psychological term) to impute missing data [37]. Chen et al. presented a multiple linear regression analysis DEA approach (regression DEA) [38].

However, the above-mentioned techniques have a few disadvantages. First, they use simple imputation methods or deletion methods to handle missing data, which may lead to erroneous results. Second, while they modify basic radial DEA models to measure the efficiency of DMUs with missing data, they are unable to deal with integer-valued variables or undesirable variables. If decision-makers simply round up the DEA solutions to the nearest integers, the results may be wrong [39–42]. Integer-valued DEA models have attracted researchers because inputs and outputs can only be integer numbers in many cases. Lozano and Villa [39], Du et al. [40], Ajirlo et al. [41], Kordrostami et al. [42], Ren et al. [43], and other scholars have applied integer-valued DEA models to many fields, e.g., universities, Olympic games, and pallet rental companies. Measuring the efficiency of DMUs with undesirable outputs is another hot topic in DEA research. There are several approaches to handle undesirable outputs, e.g., weak disposability assumption [44], direction distance function [45, 46], linear or non-linear monotonic decreasing transformation [47, 48], treating undesirable outputs as inputs [49], and applying the SBM (Slacks-Based Measure) approach and proposing additive DEA models [50].

Another disadvantage of radial DEA models is that they have weaker discriminatory ability than non-radial DEA models [51, 52]. Radial DEA models can only proportionally reduce inputs or increase outputs, while non-radial DEA models, e.g., the additive DEA [53], the enhanced Russell measure [54], and the slacks-based measure [55], do not need to make the assumption of proportional changes [56].

In this study, to handle missing data in DEA a multi-criteria evaluation approach is proposed based on the Hot deck imputation, regression imputation, Halo effect, interval DEA, integer DEA, additive DEA, DEA with undesirable outputs, and multi-index comprehensive evaluation. The main advantages of this approach are as follows. (1) The approach not only estimates the upper and lower bounds of DMUs’ efficiency but also evaluates the “relative” efficiency of these DMUs based on the “Halo + Hot deck” DEA method (if there is no correlation between variables) or regression DEA techniques (if there is a correlation between variables). Therefore, the evaluation results are relatively diverse, which avoids the shortcoming of simple imputation methods as mentioned above. (2) A multi-index comprehensive evaluation system, which involves many important factors related to the variables with missing data, is established to determine which scenario (the lower bound of efficiency, the “relative” efficiency, or the upper bound of efficiency) should be selected. The multi-index comprehensive evaluation method guarantees that the resulting efficiency is more reliable. (3) Interval additive integer-valued DEA models with undesirable outputs are proposed. These models can be used to handle integer-valued variables and undesirable outputs.

The rest of this paper is structured as follows. The multi-criteria evaluation approach (including the interval additive integer-valued DEA models with undesirable outputs and the “Hao + Hot deck” imputation method) is presented in Section 2. In Section 3, the proposed approach is applied to the pallet rental industry, and the effectiveness of the methodology is examined by analyzing error rates. Conclusions and the contributions of this paper are presented in Section 4.

## 2. Methodology

**Fig 1:**
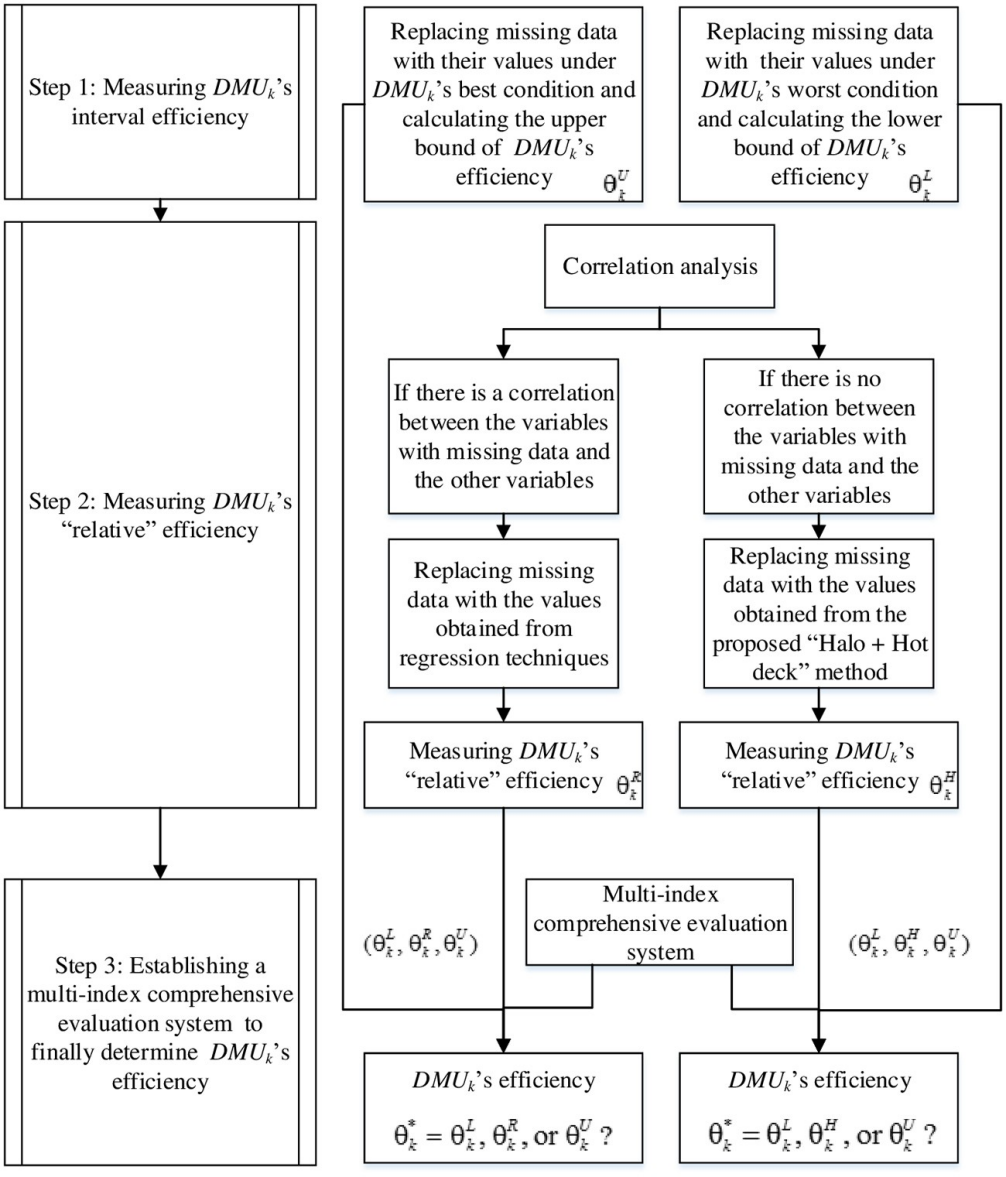
The proposed multi-criteria evaluation approach for measuring the performance of DMUs with missing data.

Assume that *Q* represents a group of DMUs. Each *DMU*_*i*_ (*DMU*_*i*_ ∈ *Q*, *i* = 1, 2, *q*) consumes *r* inputs *x*_*ji*_(*j* = 1, 2, …, *r*) to produce *m* desirable outputs *y*_*pi*_(*p* = 1, 2, …, *m*) and *t* undesirable outputs *z*_*hi*_(*h* = 1, 2, …., *t*). Further assume that the data related to some DMUs’ important variables are missing. The multi-criteria evaluation approach for measuring the performance of *DMU*_*k*_ (*DMU*_*k*_ ∈ *Q*) is shown in Fig 1, and the corresponding algorithm is as follows.

Step 1: Measuring *DMU*_*k*_’s interval efficiency.
  First, the analysts should replace missing data with their values under *DMU*_*k*_’s (the DMU under evaluation, *DMU*_*k*_ ∈ *Q*) best condition (see Subsection 2.1, model 1). Second, analysts should replace missing data with their values under *DMU*_*k*_’s worst condition (see Subsection 2.1, model 2). Then, analysts can apply the interval additive integer-valued DEA models with undesirable outputs, which is proposed in Subsection 2.1, to calculate the upper bound of *DMU*_*k*_’s efficiency ($\theta_{k}^{U}$) under *DMU*_*k*_’s best condition and the lower bound of *DMU*_*k*_’s efficiency ($\theta_{k}^{L}$) under *DMU*_*k*_’s worst condition. Therefore, *DMU*_*k*_’s interval efficiency $\left[\theta_{k}^{L},\theta_{k}^{U}\right]$ can be evaluated.
Step 2: Measuring *DMU*_*k*_’s “relative” efficiency.
  Analysts should study the relationship between the variables with missing data and the other variables. There are several methods for correlation analysis, e.g. the scatter diagram method, Pearson’s correlation coefficient, Spearman’s rank correlation coefficient, and the least squares method [57].
  If there is a correlation between variables, analysts should replace missing data with the values obtained from the regression imputation method and apply the DEA to calculate *DMU*_*k*_’s “relative” efficiency ($\theta_{k}^{R}$). Otherwise, analysts should replace missing data with the values obtained from the “Halo + Hot deck” imputation method and apply the DEA (“Halo + Hot deck” DEA) to calculate *DMU*_*k*_’s “relative” efficiency ($\theta_{k}^{H}$). The “Halo + Hot deck” imputation method is presented in Subsection 2.2. Regarding the regression imputation method, since it is well-understood, the paper does not provide a detailed explanation. As mentioned in Section 1, there are many regression techniques, so analysts should select the right regression technique based on the detailed analysis of variables, e.g., the type of variables and shape of the regression line. There must be $\theta_{k}^{L}\leq \theta_{k}^{R}\leq \theta_{k}^{U}$ or $\theta_{k}^{L}\leq \theta_{k}^{H}\leq \theta_{k}^{U}$ (see Subsection 2.2).
Step 3: Establishing a multi-index comprehensive evaluation system to finally determine *DMU*_*k*_’s efficiency.
  To finally determine *DMU*_*k*_’s efficiency ($\theta_{k}^{*}=\theta_{k}^{L},\theta_{k}^{R},\text{or}\theta_{k}^{U}?$; $\theta_{k}^{*}=\theta_{k}^{L},\theta_{k}^{H},\text{or}\theta_{k}^{U}?$) analysts should establish a multi-index comprehensive evaluation system. The indicators should be related to the variables with missing data, and the evaluation method can be qualitative or quantitative. An example is proposed in Section 3. Decisions makers can rank all DMUs after they finally determine the efficiency of all DMUs.

### 2.1 Interval additive integer-valued DEA models with undesirable outputs

Assume that some of the inputs and desirable outputs can only take integer values. Following Du et al. [40] and Ren et al. [43], *J*^*NI*^ and *J*^*I*^ respectively represent the subsets of real-valued and integer-valued inputs, while *P*^*NI*^ and *P*^*I*^ respectively denote the subsets of real-valued and integer-valued desirable outputs. Hence, *x*_*ji*_ ∈ *J*^*NI*^(*j* = 1, 2, …, *g*) and *x*_*ji*_ ∈ *J*^*I*^ (*j* = *g* + 1, *g* + 2, …, *r*) respectively imply *DMU*_*k*_’s real-valued and integer-valued inputs, while *y*_*pi*_ ∈ *P*^*NI*^ (*p* = 1, 2, …, *o*) and *y*_*pi*_ ∈ *P*^*I*^ (*p* = *o* + 1, *o* + 2, …, *m*) respectively indicate *DMU*_*k*_’s real-valued and integer-valued desirable outputs.

Model (1) and model (2), which are interval additive integer-valued DEA models with undesirable outputs, are developed to measure the upper and lower bounds of *DMU*_*k*_’s interval efficiency, respectively. Additive DEA models are proposed because they are non-radial DEA models that can distinguish all inefficiencies [53]. To deal with undesirable outputs the SBM approach is applied and additive DEA models are proposed [50].

To calculate the upper bound of *DMU*_*k*_’s efficiency (model 1), the analysts should replace missing data with their values under *DMU*_*k*_’s best condition (as stated above), which means that analysts should replace *DMU*_*k*_’s missing data related to inputs, desirable outputs, and undesirable outputs with $x_{jk}^{L}=\text{min}(all\;x_{ji}^{}\;with\;precise\;data)$, $y_{pk}^{U}=\text{max}(all\;y_{pi}^{}\;with\;precise\;data)$, and $z_{hk}^{L}=\text{min}(all\;z_{hi}^{}\;with\;precise\;data)$, respectively. If there are also some DMUs (*DMU*_*i*_ ∈ *Q*, *i* ≠ *k*) with missing data besides *DMU*_*k*_, analysts should also replace their missing data related to inputs, desirable outputs, and undesirable outputs with $x_{ji}^{U}=\text{max}(all\;x_{ji}^{}\;with\;precise\;data),i\neq k$, $y_{pi}^{L}=\text{min}(all\;y_{pi}^{}\;with\;precise\;data),i\neq k$, and $z_{hi}^{U}=\text{max}(all\;z_{hi}^{}\;with\;precise\;data),i\neq k$, respectively.

$$\begin{array}{ll} \text{max}= & \frac{1}{m+r+t}(\sum_{p=1}^{o}\frac{s_{p}{}^{+}}{y_{pk}^{U}}+\sum_{p=o+1}^{m}\frac{s_{p}{}^{I+}}{y_{pk}^{U}}+\sum_{j=1}^{g}\frac{s_{j}{}^{-}}{x_{jk}^{L}}+\sum_{j=g+1}^{r}\frac{s_{j}{}^{I-}}{x_{jk}^{L}}+\sum_{h=1}^{t}\frac{s_{h}{}^{-}}{z_{hk}^{L}})\\ s.t. & \sum_{i=1,i\neq k}^{q}x_{ji}^{U}\lambda_{i}\text{+}x_{jk}^{L}\lambda_{k}\text{+}s_{j}{}^{-}=x_{jk}^{L},j=1,2,\ldots ,g\\ & \sum_{i=1,i\neq k}^{q}x_{ji}^{U}\lambda_{i}\text{+}x_{jk}^{L}\lambda_{k}\leq {\tilde{x}}_{jk},j=g+1,g+2,\ldots ,r\\ & x_{jk}^{L}-s_{j}{}^{I-}={\tilde{x}}_{jk},j=g+1,g+2,\ldots ,r\\ & \sum_{i=1,i\neq k}^{q}y_{pi}^{L}\lambda_{i}+y_{pk}^{U}\lambda_{k}-s_{p}{}^{+}=y_{pk}^{U},p=1,2,\ldots ,o\\ & \sum_{i=1,i\neq k}^{q}y_{pi}^{L}\lambda_{i}+y_{pk}^{U}\lambda_{k}\geq {\tilde{y}}_{pk},p=o+1,o+2\ldots ,m\\ & y_{pk}^{U}+s_{p}{}^{I+}={\tilde{y}}_{pk},p=o+1,o+2\ldots ,m\\ & \sum_{i=1,i\neq k}^{q}z_{hi}^{U}\lambda_{i}+z_{hk}^{L}\lambda_{k}\text{+}s_{h}{}^{-}=z_{hk}^{L},h=1,2,\ldots ,t\\ & \sum_{i=1}^{q}\lambda_{i}=1\\ & \lambda_{i}\geq 0,i=1,2,\ldots ,q\\ & s_{j}{}^{-}\geq 0,j=1,2,\ldots ,g\\ & s_{j}{}^{I-}\geq 0,j=g+1,g+2,\ldots ,r\\ & s_{p}{}^{+}\geq 0,p=1,2,\ldots ,o\\ & s_{p}{}^{I+}\geq 0,p=o+1,o+2\ldots ,m\\ & s_{h}{}^{-}\geq 0,h=1,2,\ldots ,t\\ & {\tilde{x}}_{jk}\in J^{I},j=g+1,g+2,\ldots ,r\\ & {\tilde{y}}_{pk}\in P^{I},p=o+1,o+2,\ldots ,m \end{array}$$ (1)

where *λ*_*i*_ indicates the weight for *DMU*_*i*_; $s_{j}{}^{-}$, $s_{j}{}^{I-}$, $s_{p}{}^{+}$, $s_{p}{}^{I+}$, and $s_{h}{}^{-}$ respectively represent the slack variables for real-valued inputs, integer-valued inputs, real-valued desirable outputs, integer-valued desirable outputs, and undesirable outputs, respectively; ${\tilde{x}}_{jk}\left(j=g+1,g+2,\ldots ,r\right)$ and ${\tilde{y}}_{pk}\left(p=o+1,o+2\ldots ,m\right)$ are the targets for integer-valued inputs and integer-valued desirable outputs, respectively. Note that the superscript “*U*” and “*L*” respectively indicate the upper bound and lower bound values of the related variables.

To calculate the lower bound of *DMU*_*k*_’s efficiency (model 2), the analysts should replace missing data with their values under *DMU*_*k*_’s worst condition (as stated above), which means that analysts should replace *DMU*_*k*_’s missing data related to inputs, desirable outputs, and undesirable outputs with $x_{jk}^{U}=\text{max}(all\;x_{ji}^{}\;with\;precise\;data)$, $y_{pk}^{L}=\text{min}(all\;y_{pi}^{}\;with\;precise\;data)$, and $z_{hk}^{U}=\text{max}(all\;z_{hi}^{}\;with\;precise\;data)$, respectively. If there are also some DMUs (*DMU*_*i*_ ∈ *Q*, *i* ≠ *k*) with missing data besides *DMU*_*k*_, as discussed above, analysts should also respectively replace their missing data related to inputs, desirable outputs, and undesirable outputs with $x_{ji}^{L}=\text{min}(all\;x_{ji}^{}\;with\;precise\;data),i\neq k$, $y_{pi}^{U}=\text{max}(all\;y_{pi}^{}\;with\;precise\;data),i\neq k$, and $z_{hi}^{L}=\text{max}(all\;z_{hi}^{}\;with\;precise\;data),i\neq k$.

$$\begin{array}{ll} \text{max}= & \frac{1}{m+r+t}(\sum_{p=1}^{o}\frac{s_{p}{}^{+}}{y_{pk}^{L}}+\sum_{p=o+1}^{m}\frac{s_{p}{}^{I+}}{y_{pk}^{L}}+\sum_{j=1}^{g}\frac{s_{j}{}^{-}}{x_{jk}^{U}}+\sum_{j=g+1}^{r}\frac{s_{j}{}^{I-}}{x_{jk}^{U}}+\sum_{h=1}^{t}\frac{s_{h}{}^{-}}{z_{hk}^{U}})\\ s.t. & \sum_{i=1,i\neq k}^{q}x_{ji}^{L}\lambda_{i}\text{+}x_{jk}^{U}\lambda_{k}\text{+}s_{j}{}^{-}=x_{jk}^{U},j=1,2,\ldots ,g\\ & \sum_{i=1,i\neq k}^{q}x_{ji}^{L}\lambda_{i}\text{+}x_{jk}^{U}\lambda_{k}\leq {\tilde{x}}_{jk},j=g+1,g+2,\ldots ,r\\ & x_{jk}^{U}-s_{j}{}^{I-}={\tilde{x}}_{jk},j=g+1,g+2,\ldots ,r\\ & \sum_{i=1,i\neq k}^{q}y_{pi}^{U}\lambda_{i}+y_{pk}^{L}\lambda_{k}-s_{p}{}^{+}=y_{pk}^{L},p=1,2,\ldots ,o\\ & \sum_{i=1,i\neq k}^{q}y_{pi}^{U}\lambda_{i}+y_{pk}^{L}\lambda_{k}\geq {\tilde{y}}_{pk},p=o+1,o+2\ldots ,m\\ & y_{pk}^{L}+s_{p}{}^{I+}={\tilde{y}}_{pk},p=o+1,o+2\ldots ,m\\ & \sum_{i=1,i\neq k}^{q}z_{hi}^{L}\lambda_{i}+z_{hk}^{U}\lambda_{k}\text{+}s_{h}{}^{-}=z_{hk}^{U},h=1,2,\ldots ,t\\ & \sum_{i=1}^{q}\lambda_{i}=1\\ & \lambda_{i}\geq 0,i=1,2,\ldots ,q\\ & s_{j}{}^{-}\geq 0,j=1,2,\ldots ,g\\ & s_{j}{}^{I-}\geq 0,j=g+1,g+2,\ldots ,r\\ & s_{p}{}^{+}\geq 0,p=1,2,\ldots ,o\\ & s_{p}{}^{I+}\geq 0,p=o+1,o+2\ldots ,m\\ & s_{h}{}^{-}\geq 0,h=1,2,\ldots ,t\\ & {\tilde{x}}_{jk}\in J^{I},j=g+1,g+2,\ldots ,r\\ & {\tilde{y}}_{pk}\in P^{I},p=o+1,o+2,\ldots ,m \end{array}$$ (2)

The mathematical notations used in model (2) are the same as those used in model (1). Different from traditional additive DEA models, both model (1) and model (2) are unit-invariant [58]. Model (1) and model (2) cannot provide the efficiency scores, so Eqs (3) and (4) are proposed to calculate the upper bound and lower bound of *DMU*_*k*_’s efficiency, respectively.

$$\theta_{k}^{U}=\frac{1-\frac{1}{r}(\sum_{j=1}^{g}s_{j}{}^{-}*/x_{jk}^{L}+\sum_{j=g+1}^{r}s_{j}{}^{I-}*/x_{jk}^{L})}{1+\frac{1}{m+t}(\sum_{p=1}^{o}s_{p}{}^{+}*/y_{pk}^{U}+\sum_{p=o+1}^{m}s_{p}{}^{I+}*/y_{pk}^{U}+\sum_{h=1}^{t}s_{h}{}^{-}*/z_{hk}^{L})}$$ (3)

in which $\left\{\lambda_{i}*,s_{j}{}^{-}*,s_{j}{}^{I-}*,s_{p}{}^{+}*,s_{p}{}^{I+}*,s_{h}{}^{-}*,{\tilde{x}}_{jk}*,{\tilde{y}}_{pk}*\right\}$ is the optimum solution resulting from model (1). There must be $0\leq \theta_{k}^{U}\leq 1$. $\theta_{k}^{U}=1$ implies that *DMU*_*k*_ is additive-efficient under *DMU*_*k*_’s best condition because $\theta_{k}^{U}$ equals to 1 if and only if all slacks variables are equal to 0. The greater value of $\theta_{k}^{U}$, the better performance of *DMU*_*k*_.

$$\theta_{k}^{L}=\frac{1-\frac{1}{r}(\sum_{j=1}^{g}s_{j}{}^{-}*/x_{jk}^{U}+\sum_{j=g+1}^{r}s_{j}{}^{I-}*/x_{jk}^{U})}{1+\frac{1}{m+t}(\sum_{p=1}^{o}s_{p}{}^{+}*/y_{pk}^{L}+\sum_{p=o+1}^{m}s_{p}{}^{I+}*/y_{pk}^{L}+\sum_{h=1}^{t}s_{h}{}^{-}*/z_{hk}^{U})}$$ (4)

in which $\left\{\lambda_{i}*,s_{j}{}^{-}*,s_{j}{}^{I-}*,s_{p}{}^{+}*,s_{p}{}^{I+}*,s_{h}{}^{-}*,{\tilde{x}}_{jk}*,{\tilde{y}}_{pk}*\right\}$ is the optimum solution resulting from model (2). There must be also $0\leq \theta_{k}^{L}\leq 1$. $\theta_{k}^{L}=1$ implies that *DMU*_*k*_ is additive-efficient under *DMU*_*k*_’s worst condition. The greater value of $\theta_{k}^{L}$, the better performance of *DMU*_*k*_.

### 2.2 “Halo + Hot deck” imputation method

#### 2.2.1 Halo effect

Halo effect is a psychological term proposed by Thorndike in 1920 [59]. It means that an individual’s positive thoughts about a company (person, product, brand, and so on) in one area positively affect how he/she thinks of the company in other areas [60]. This theory can be applied to evaluate DMUs’ relative efficiency. If *DMU*_*k*_’s relative efficiency ($\theta_{k}^{N}$) is better than that of other DMUs’ when not taking into account the variables with missing data (deleting the variables with missing data when measuring the performance of DMUs), it can be thought that this DMU’s relative efficiency ($\theta_{k}^{*}$) would also be better when taking into account the variables with missing data. Model (1) and Eq (3) (or model 2 and Eq 4) can be applied to calculate $\theta_{k}^{N}$ by deleting all the symbols related to the variables with missing data. However, the Halo effect may lead to bias. To overcome this shortcoming, we propose the multi-criteria evaluation approach (See Fig 1).

#### 2.2.2 “Halo + Hot deck”

According to the Hot deck imputation method, as mentioned in Section 1, the missing data should be replaced with the observed values from a “similar” unit. Therefore, based on the ideas of the Halo effect and Hot deck imputation, the missing data related to *DMU*_*k*_ can be replaced with the values of a DMU with “similar efficiency $\theta_{k}^{N}$”. The “Halo + Hot deck” imputation method is as follows.

Based on the relative efficiency of all DMUs without considering the variables with missing data, a “similar” DMU whose relative efficiency is less than *DMU*_*k*_’s efficiency and a “similar” DMU whose relative efficiency is greater than *DMU*_*k*_‘s efficiency can be found. Then, the missing data about *DMU*_*k*_ can be replaced with the average of the two “similar” DMUs’ related values. The missing data related to *DMU*_*k*_ are not replaced with the values of the “closest” DMU because it may lead to larger errors. Note that there may be several DMUs that have the same efficiency scores as *DMU*_*k*_‘s. In that case, analysts can just replace the missing data about *DMU*_*k*_ with the average of these “same” DMUs’ related values.

#### 2.2.3 Measuring the “relative” efficiency

Model (1) and Eq (3) (or model 2 and Eq 4) can be applied to calculate the “relative” efficiency $\theta_{k}^{H}$ based on the “Halo + Hot deck” imputation method (this method is called “Halo + Hot deck” DEA which means the “Halo + Hot deck” imputation method + the DEA approach), but analysts should set $x_{jk}^{L}=x_{jk}^{H}$ as well as $x_{ji}^{U}=x_{ji}^{H}(i\neq k)$ (or $x_{jk}^{U}=x_{jk}^{H}$ as well as $x_{ji}^{L}=x_{ji}^{H}(i\neq k)$), $z_{hk}^{L}=z_{hk}^{H}$ as well as $z_{hi}^{U}=z_{hi}^{H}(i\neq k)$ (or $z_{hk}^{U}=z_{hk}^{H}$ as well as $z_{hi}^{L}=z_{hi}^{H}(i\neq k)$), and $y_{pk}^{U}=y_{pk}^{H}$ as well as $y_{pi}^{L}=y_{pi}^{H}(i\neq k)$ (or $y_{pk}^{L}=y_{pk}^{H}$ as well as $y_{pi}^{U}=y_{pi}^{H}(i\neq k)$). The superscript *H* indicates that the values of the variables with missing data are obtained from the “Halo + Hot deck” imputation method.

There must be $\theta_{k}^{L}\leq \theta_{k}^{H}\leq \theta_{k}^{U}$ because there are $x_{ji}^{L}\leq x_{ji}^{H}\leq x_{ji}^{U}$, $y_{pi}^{L}\leq y_{pi}^{H}\leq y_{pi}^{U}$, and $z_{hi}^{L}\leq z_{hi}^{H}\leq z_{hi}^{U}$ for *i* = 1, 2, …*q*. Similarly, there must be $\theta_{k}^{L}\leq \theta_{k}^{R}\leq \theta_{k}^{U}$ because there are $x_{ji}^{L}\leq x_{ji}^{R}\leq x_{ji}^{U}$, $y_{pi}^{L}\leq y_{pi}^{R}\leq y_{pi}^{U}$, and $z_{hi}^{L}\leq z_{hi}^{R}\leq z_{hi}^{U}$ for *i* = 1, 2, …*q*. The superscript *R* indicates that the values of the variables with missing data are obtained from regression imputation methods.

## 3. Numerical illustrations

This section applies the proposed approach to analyze the efficiency of pallet rental companies. There is limited quantitative research in the pallet rental industry because the data related to this industry are not publicly available [61, 62]. Therefore, it is necessary to propose an approach to evaluate the performance of pallet rental companies when some important data are missing, and this research is important to the pallet rental industry. Also, this industry involves undesirable outputs, e.g., pallet loss, and some of the inputs are integer numbers. The proposed approach is able to deal with these types of data.

### 3.1 Data

There are twelve pallet rental companies in the dataset including Commonwealth Handling Equipment Pool (CHEP), Intelligent Global Pooling Systems (iGPS), PECO Pallet, H & H Pallet Leasing, La Palette Rouge (LPR), Pooling Partner, Contraloadad, Nippon Pallet Pool System, Japan Pallet Rental (JPR), Korea Pallet Pool (KPP), Loscam, and Jituo Pallet Pool. Each company uses two integer-valued inputs (employees *x*_1*i*_ and pallets *x*_2*i*_) to produce one real-valued desirable output (annual revenue *y*_1*i*_) and one real-valued undesirable output (annual pallet loss rate *z*_1*i*_), and the data related to these companies in 2018 are shown in Table 1 [43, 62]. The data about *x*_1*i*_, *x*_2*i*_, and *y*_1*i*_ (unit: million U.S. dollars) are obtained from the official websites of these companies as well as other relevant websites, and the values of *z*_1*i*_(unit: percent) are estimated by managers in these companies. Model (1) and Eq (3) (or model 2 and Eq 4) can be applied to evaluate the efficiency of these companies using these precise data, and the resulting efficiency ($\theta_{i}^{P}$) is precise. The results are also shown in Table 1. Note that analysts should set $x_{1i}^{U}=x_{1i}^{L}=x_{1i}^{}$, $x_{2i}^{U}=x_{2i}^{L}=x_{2i}^{}$,$y_{1i}^{U}=y_{1i}^{L}=y_{1i}^{}$, and $z_{1i}^{U}=z_{1i}^{L}=z_{1i}^{}$ for *i* = 1, 2, …, 12.

**Table 1:** Variables and the precise efficiency.

DMU	*x*_1i_	*x*_2i_	*y*_1i_	*z*_1i_	$\theta_{i}^{P}$
1	239	10000000	85.34	2	1.000
2	7500	460000000	4048.30	2	1.000
3	310	92000000	248.60	8	0.189
4	875	40000000	370.72	10	0.272
5	284	9600000	226.03	1	1.000
6	130	3000000	45.21	12	0.346
7	101	3000000	58.07	10	0.520
8	175	10000000	75.00	12	0.240
9	144	8000000	49.90	10	0.218
10	16	22500	1.97	12	1.000
11	109	7000000	313.80	8	1.000
12	20	5000	1.50	12	1.000
Ave.	825.25	53552291.67	460.37	8.25	0.649
Max.	7500.00	460000000.00	4048.30	12.00	1
Min.	16.00	5000.00	1.50	1.00	0.189
Std. Dev.	2114.20	130618453.80	1136.78	4.22	0.384

To apply the proposed multi-criteria evaluation approach to this case, it is assumed that the data about some DMUs’ annual pallet loss rates are missing ($z_{1i}^{M}$). Note that $z_{1i}^{M}$ represents missing data while *z*_1*i*_ indicates precise data. Twelve scenarios (*l* = 1, 2, …, 12) are considered. Scenario *l* indicates that the value of *DMU*_*i*_’s annual pallet loss rate is missing. For example, Scenario 4 represents that the value of DMU 4’s annual pallet loss rate is missing. Then, the proposed approach can be applied to measure the efficiency of all companies ($\theta_{i}^{*}$).

The effectiveness of the proposed approach can be estimated by the error rate *ε* that can be calculated by $\varepsilon =\frac{\sum_{l=1}^{12}\varepsilon_{l}}{\text{12}}$ where $\varepsilon_{l}=\sum_{i=1}^{12}\frac{\left|\theta_{i}^{*}-\theta_{i}^{P}\right|}{\theta_{i}^{P}}$. The lower the value of *ε* is, the better the performance of the approach should be.

### 3.2 Measuring the efficiency of pallet rental companies using the proposed multi-criteria evaluation approach

In this subsection, the proposed approach is applied to measure the efficiency of the twelve companies.

#### 3.2.1 Interval efficiency

As stated in Section 2, analysts should first measure *DMU*_*k*_’s interval efficiency. The lower and upper bounds of DMU 5’s annual pallet loss rate are 2 and 12, respectively, while the lower and upper bounds of the other DMUs’ annual pallet loss rates are all 1 and 12, respectively. Tables 2 and 3 show the interval efficiency resulting from the proposed interval additive integer-valued DEA models with undesirable outputs (model 1 as well as Eq 3 and model 2 as well as Eq 4).

**Table 2:** Efficiency resulting from the interval approach (DMU 1-DMU 6).

Scenario	DMU 1	DMU 2	DMU 3	DMU 4	DMU 5	DMU 6
1-U	1.000	1.000	0.189	0.272	1.000	0.346
1-H	0.234	1.000	0.189	0.272	1.000	0.346
1-L	0.231	1.000	0.189	0.272	1.000	0.346
2-U	1.000	1.000	0.189	0.272	1.000	0.346
2-H	1.000	1.000	0.189	0.274	1.000	0.346
2-L	1.000	1.000	0.189	0.274	1.000	0.346
3-U	1.000	1.000	1.000	0.272	1.000	0.346
3-H	1.000	1.000	0.169	0.272	1.000	0.346
3-L	1.000	1.000	0.165	0.272	1.000	0.346
4-U	1.000	1.000	0.189	1.000	1.000	0.346
4-H	1.000	1.000	0.189	0.264	1.000	0.346
4-L	1.000	1.000	0.189	0.257	1.000	0.346
5-U	1.000	1.000	0.189	0.272	1.000	0.346
5-H	1.000	1.000	0.189	0.272	0.560	0.346
5-L	1.000	1.000	0.189	0.272	0.409	0.346
6-U	0.501	1.000	0.189	0.272	1.000	1.000
6-H	1.000	1.000	0.189	0.272	1.000	0.575
6-L	1.000	1.000	0.189	0.272	1.000	0.346
7-U	0.491	1.000	0.189	0.272	1.000	0.346
7-H	1.000	1.000	0.189	0.272	1.000	0.346
7-L	1.000	1.000	0.189	0.272	1.000	0.346
8-U	0.556	1.000	0.189	0.272	1.000	0.346
8-H	1.000	1.000	0.189	0.272	1.000	0.346
8-L	1.000	1.000	0.189	0.272	1.000	0.346
9-U	0.543	1.000	0.189	0.272	1.000	0.346
9-H	1.000	1.000	0.189	0.272	1.000	0.346
9-L	1.000	1.000	0.189	0.272	1.000	0.346
10-U	0.485	1.000	0.189	0.272	1.000	0.307
10-H	1.000	1.000	0.189	0.272	1.000	0.346
10-L	1.000	1.000	0.189	0.272	1.000	0.346
11-U	0.223	1.000	0.136	0.207	0.466	0.327
11-H	1.000	1.000	0.179	0.261	1.000	0.343
11-L	1.000	1.000	0.401	0.450	1.000	0.359
12-U	0.497	1.000	0.189	0.272	1.000	0.314
12-H	1.000	1.000	0.189	0.272	1.000	0.331
12-L	1.000	1.000	0.189	0.272	1.000	0.346

**Table 3:** Efficiency resulting from the interval approach (DMU 7-DMU 12).

Scenario	DMU 7	DMU 8	DMU 9	DMU 10	DMU 11	DMU 12
1-U	0.520	0.240	0.218	1.000	1.000	1.000
1-H	0.520	0.240	0.218	1.000	1.000	1.000
1-L	0.520	0.240	0.218	1.000	1.000	1.000
2-U	0.520	0.240	0.218	1.000	1.000	1.000
2-H	0.520	0.240	0.218	1.000	1.000	1.000
2-L	0.520	0.240	0.218	1.000	1.000	1.000
3-U	0.520	0.240	0.218	1.000	1.000	1.000
3-H	0.520	0.240	0.218	1.000	1.000	1.000
3-L	0.520	0.240	0.218	1.000	1.000	1.000
4-U	0.520	0.240	0.218	1.000	1.000	1.000
4-H	0.520	0.240	0.218	1.000	1.000	1.000
4-L	0.520	0.240	0.218	1.000	1.000	1.000
5-U	0.531	0.240	0.218	1.000	1.000	1.000
5-H	0.557	0.240	0.218	1.000	1.000	1.000
5-L	0.557	0.240	0.218	1.000	1.000	1.000
6-U	0.481	0.240	0.218	1.000	1.000	1.000
6-H	0.498	0.240	0.218	1.000	1.000	1.000
6-L	0.520	0.240	0.218	1.000	1.000	1.000
7-U	1.000	0.240	0.218	1.000	1.000	1.000
7-H	1.000	0.240	0.218	1.000	1.000	1.000
7-L	0.448	0.240	0.218	1.000	1.000	1.000
8-U	0.520	1.000	0.218	1.000	1.000	1.000
8-H	0.520	0.491	0.218	1.000	1.000	1.000
8-L	0.520	0.240	0.218	1.000	1.000	1.000
9-U	0.520	0.240	1.000	1.000	1.000	1.000
9-H	0.520	0.240	0.263	1.000	1.000	1.000
9-L	0.520	0.240	0.214	1.000	1.000	1.000
10-U	0.396	0.240	0.218	1.000	1.000	1.000
10-H	0.434	0.240	0.218	1.000	1.000	1.000
10-L	0.520	0.240	0.218	1.000	1.000	1.000
11-U	0.432	0.217	0.199	1.000	1.000	1.000
11-H	0.466	0.236	0.215	1.000	1.000	1.000
11-L	0.672	0.255	0.275	1.000	1.000	1.000
12-U	0.406	0.240	0.218	1.000	1.000	1.000
12-H	0.439	0.240	0.218	1.000	1.000	1.000
12-L	0.520	0.240	0.218	1.000	1.000	1.000

In Tables 2 and 3, the sub-scenario *l*-U, the sub-scenario *l*-H, and the sub-scenario *l*-L represent the efficiency of these companies under *DMU*_*k*_ ‘s best condition, “Halo + Hot deck” condition, and worst condition, respectively. Therefore, the efficiency of *DMU*_*k*_ in the three sub-scenarios is indicated by $\theta_{k}^{U},\theta_{k}^{H},\theta_{k}^{L}$, respectively.

Note that the “Halo + Hot deck” DEA efficiency of *DMU*_*k*_ ($\theta_{k}^{H}$) is also shown in Tables 2 and 3 for the sake of clarity. X indicates the efficiency of the *DMU*_*k*_ under estimation, and X represents the efficiency of *DMU*_*i*_(*i* ≠ *k*) that changes with different values of *DMU*_*k*_’s missing data. DMU 2, DMU 10, DMU 11, and DMU 12 are fully efficient because their efficiency scores are equal to 1 in all scenarios. All DMUs are efficient under their own best condition. The value of DMU 2’s annual pallet loss rate does not affect the ranking of these companies. Thus, analysts do not need to further evaluate the “relative” efficiency of these DMUs in Scenario 2. The values of some DMUs’ annual pallet loss rates (i.e., DMU 2, DMU 5, DMU 6, DMU 7, DMU 8, DMU 9, DMU 10, DMU 11, DMU 12) are very important because they have effects on other DMUs’ efficiency. For example, the values of DMU 11’s annual pallet loss rate can affect the efficiency of DMU 1, DMU 3, DUMU 4, DMU 5, DMU 6, DMU 7, DMU 8, and DMU 9. If a DMU’s efficiency can be affected by another DMU, its efficiency score would decrease when the value of that DMU’s annual pallet loss rate decreases. Therefore, the “annual pallet loss rate” is an important variable for measuring the efficiency of pallet rental companies, and analysts should consider it when measuring efficiency.

#### 3.2.2 Measuring the “relative” efficiency

**Fig 2:**
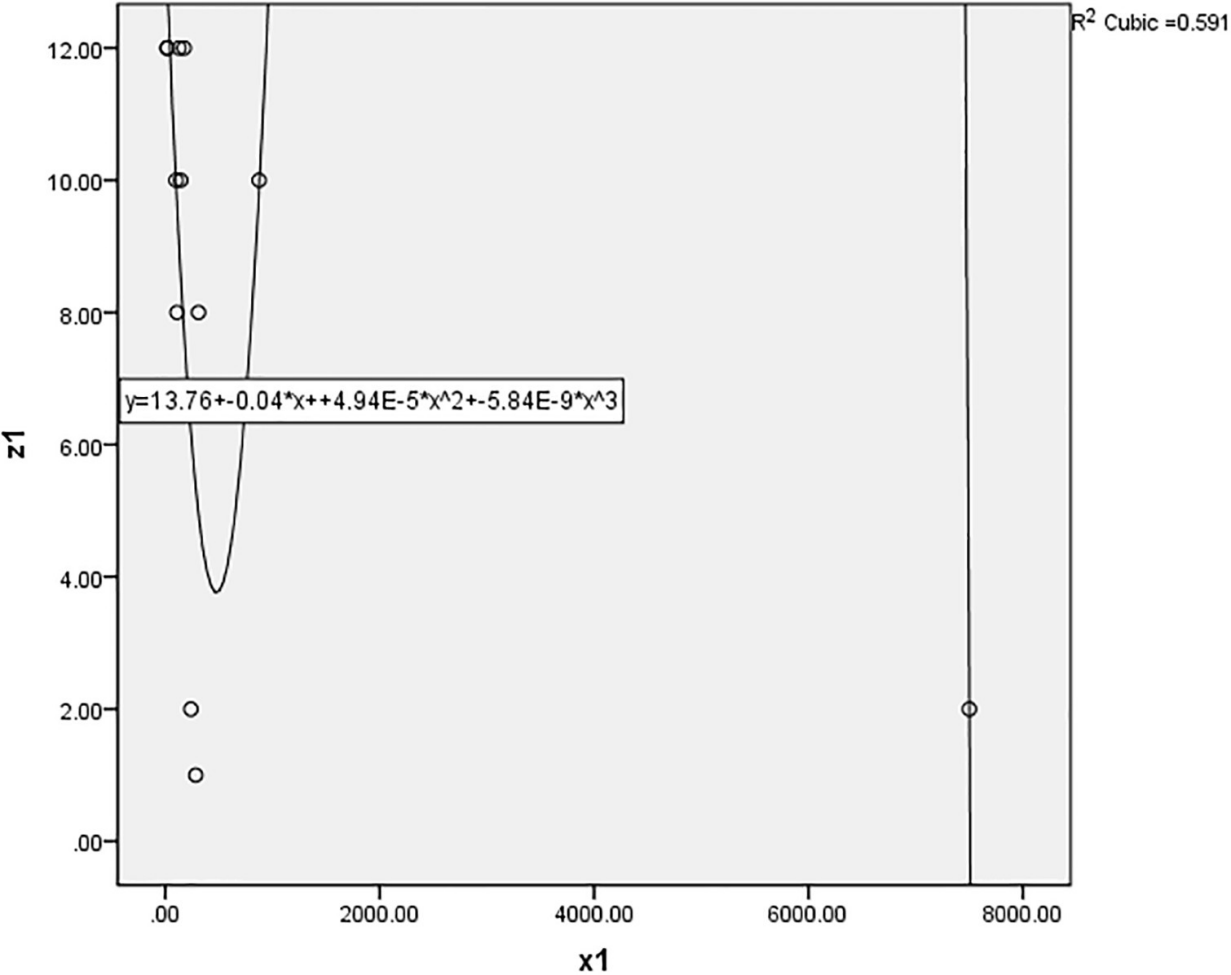
The relationship between the number of “employees” and the “annual pallet loss rate”.

**Fig 3:**
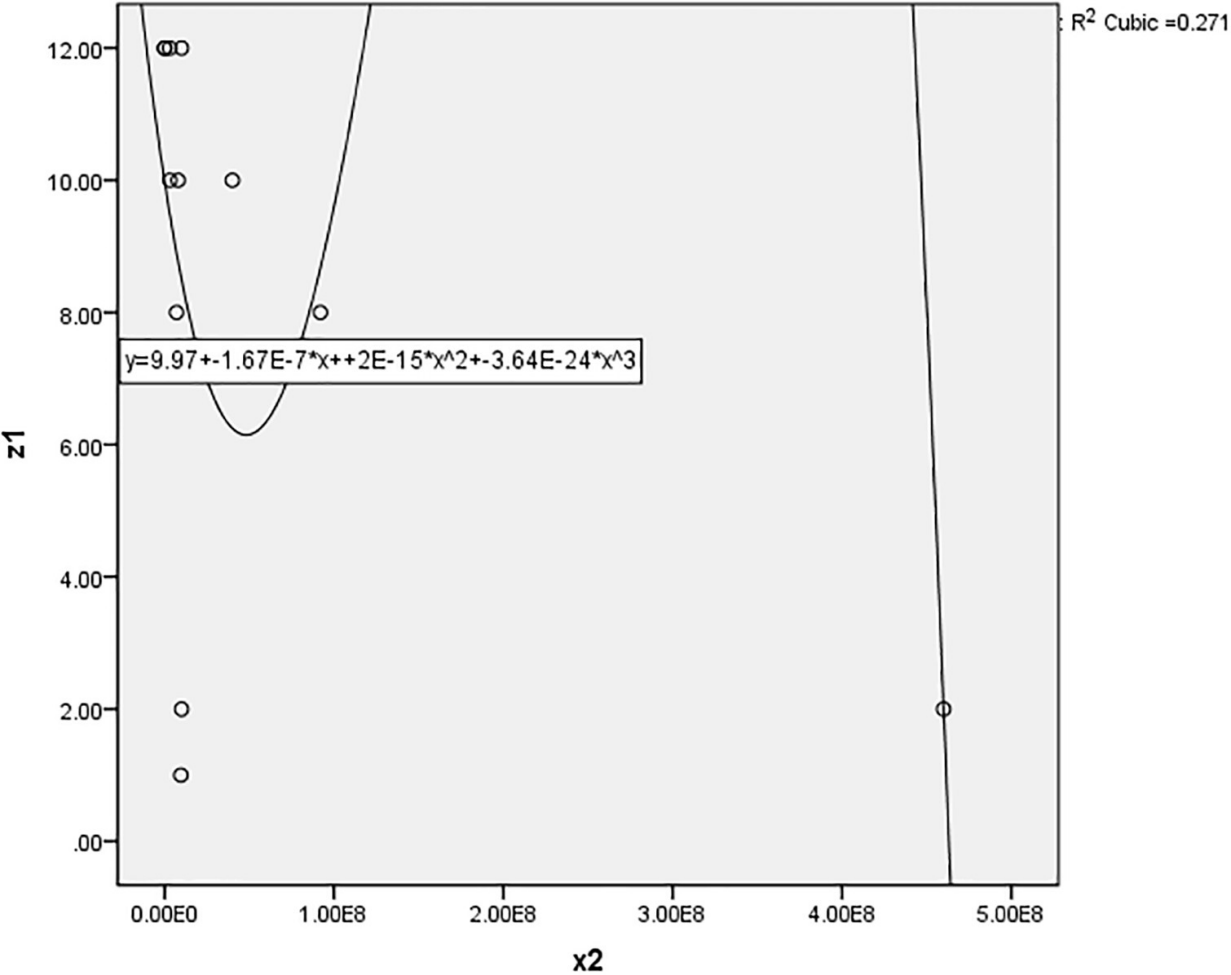
The relationship between the number of “pallets” and the “annual pallet loss rate”.

**Fig 4:**
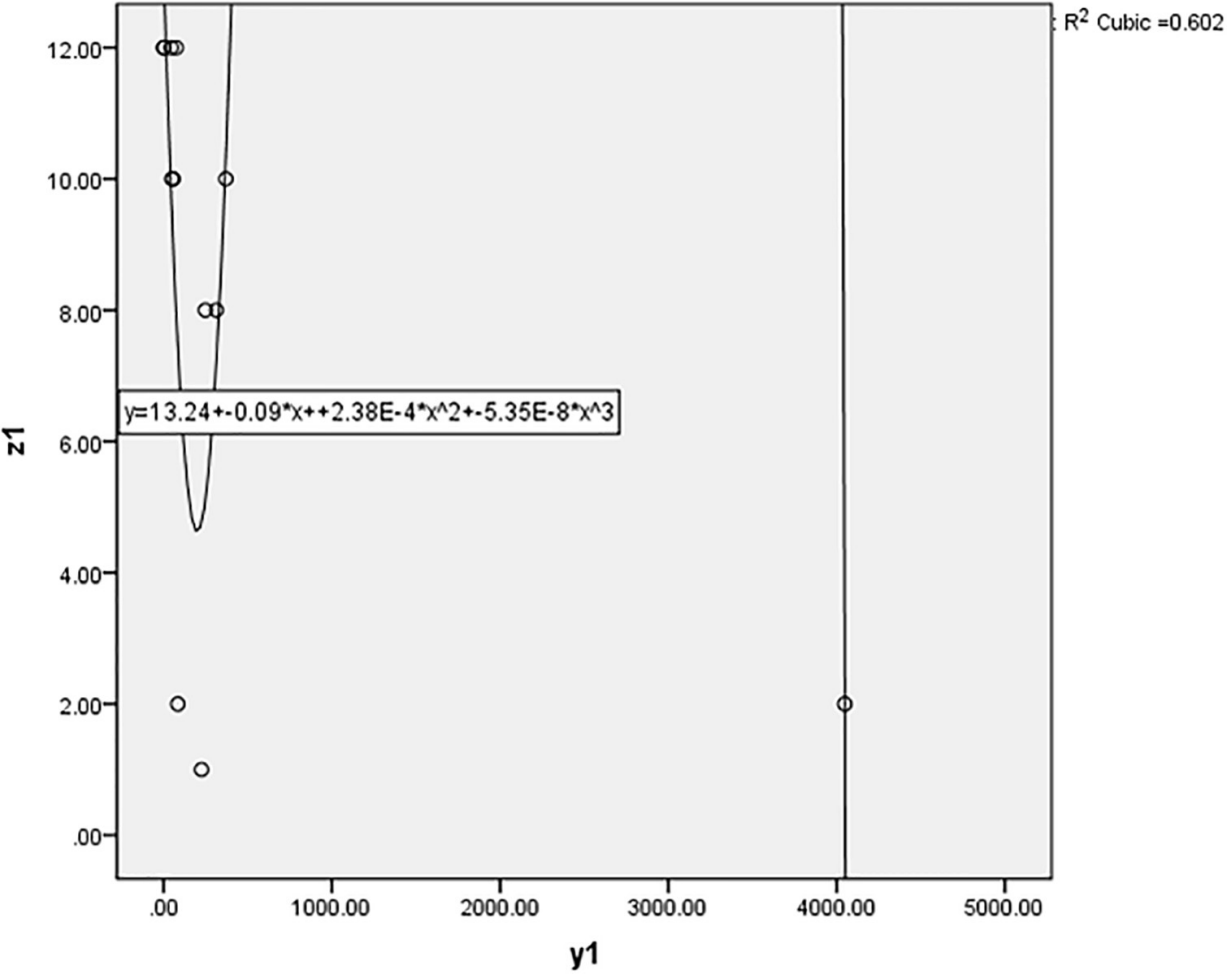
The relationship between the “annual revenue” and the “annual pallet loss rate”.

The scatter diagram method is applied to analyze the relationship between the annual pallet loss rate and the other variables. SPSS software is used to draw scatter diagrams. The results are shown in Figs 2, 3 and 4. If the R-square is greater than 0.8, there is a relationship between variables. In the case study, all the R-squares are less than 0.8, so there is no relationship between the annual pallet loss rate and the other variables. It is worth noting that the outliers (the values of DMU 2) have been removed from the diagrams and the results also show that there is no relationship between the annual pallet loss rate and the other variables. In fact, Pearson’s correlation coefficient and Spearman’s rank correlation coefficient are also applied to analyze the relationship between variables, and the results are the same. Therefore, missing data should be replaced with the values obtained from the proposed “Halo + Hot deck” imputation method.

Model (1) and Eq (3) are modified (deleting all symbols related to undesirable outputs) and applied to measure the relative efficiency ($\theta_{k}^{N}$) of all DMUs without considering the variable with missing data (the annual pallet loss rate). Table 4 shows the results.

**Table 4:** Efficiency when deleting the variable with missing data.

DMU	Efficiency	Ranking
1	0.157	11
2	1.000	1
3	0.169	9
4	0.301	7
5	0.401	5
6	0.239	8
7	0.334	6
8	0.158	10
9	0.130	12
10	1.000	1
11	1.000	1
12	1.000	1

Table 5 shows the values of $z_{1i}^{H}$ obtained from the “Halo + Hot deck” imputation method. $z_{1i}^{M}$ is replaced with $z_{1i}^{H}$. The annual pallet loss rates of DMU 3’s “similar” DMUs, i.e., DMU 6 and DMU 8, are the same so that it is needed to employ another DMU (DMU 4). There are four DMUs rank No. 1 so that DMU 5’s “similar” DMUs include five DMUs, i.e., DMU 2, DMU 7, DMU 10, DMU 11, and DMU 12. DMU 9 ranks the 12th, so its missing data should be replaced with the average of the annual pallet loss rates of DMU 1 and DMU 8. Model (1) and Eq (3) are used to measure the “relative” efficiency $\theta_{k}^{H}$ of all companies and the results are shown in Tables 2 and 3.

**Table 5:** Results obtained from the “Halo + Hot Deck” imputation.

DMU	$z_{1i}^{H}$	The interval of $z_{1i}^{M}$	The “similar” DMUs
1	11.00	[10, 12]	(DMU 8, DMU9)
2	10.00	[8, 12]	(DMU 10, DMU 11, DMU 12)
3	11.00	[10,12]	(DMU 4, DMU 6, DMU 8)
4	11.00	[10, 12]	(DMU 6, DMU 7)
5	7.00	[2, 12]	(DMU 2, DMU7, DMU 10, DMU11, DMU12)
6	9.00	[8, 10]	(DMU 3, DMU 4)
7	5.50	[1, 10]	(DMU 4, DMU 5)
8	5.00	[2, 8]	(DMU 1, DMU 3)
9	7.00	[2, 12]	(DMU 1, DMU 8)
10	7.00	[2, 12]	(DMU 2, DMU 11, DMU 12)
11	7.00	[2, 12]	(DMU 2, DMU 10, DMU12)
12	7.00	[2, 12]	(DMU 2, DMU 10, DMU 11)

#### 3.2.3 Establishing a multi-index comprehensive evaluation system to finally determine the efficiency of these pallet rental companies

The annual pallet loss rate can be affected by many factors. Experts who have researched the pallet rental industry for more than three years in the United States, the United Kingdom, and China were reviewed. They proposed the following multi-index comprehensive evaluation system to determine the values of the annual pallet loss rate (as shown in Table 6). “Experience” indicates how long a company has operated. The longer a company has operated, the better its performance would be in reducing the annual pallet loss rate. For example, if a company has operated for over 50 years, it can be regarded as the most experienced in reducing the pallet loss rate. Thus, this company’s score in the indicator “Experience” is 10. “Information management technology” indicates the level of a pallet rental company using MIS (basic management information system), barcode, RFID (radio-frequency identification), PTS (pallets tracking system), and other techniques. If a company has applied all these techniques to control pallets, this company’s score in the indicator “Information management technology” is 10. It means that the company has applied the most advanced information management technologies to reduce its pallet loss rate. “Team” represents a company’s investments in human resources for reducing the pallet loss rate. “Non-professional team” means that the company has invested in human resources but there is not a professional team that dedicates to reduce the annual pallet loss rate, so its score in the indicator “Team” is 5. “Process improvement” indicates the level of a company’s control of its business. If a company utilizes 6 sigma (6*σ*), i.e., the highest level, as standard practice, its score in the indicator “Process improvement” is 10.

**Table 6:** Multi-index comprehensive evaluation system.

Indicator	Scoring criteria
Experience	Below 1, 0;1–10, 2; 10–20, 4; 20–30, 6; 30–50, 8; over 50, 10
Information management technology	None, 0; MIS 2.5, MIS + Barcode, 5; MIS + Barcode + RFID, 7.5; MIS + Barcode + RFID + PTS + others, 10
Team	None, 0; Non-professional team, 5; Professional team, 10
Process improvement	None, 0; 3*σ*, 5; 6*σ*, 10

The group of experts was asked to score each pallet rental company based on the multi-index comprehensive evaluation system. The results are shown in Table 7. Note that these experts did not know these companies’ precise annual pallet loss rates. If the score of a company is below 20 (below 50% of the total score), this company is under the worst condition (DMU 6, DMU 7, DMU 8, DMU 9, DMU 10, and DMU 12). If the score of a company is between 20 and 32 (50%-80% of the total score), this company is under the “Halo + Hot deck” condition (DMU 3, DMU 4, and DMU 11). If the score of a company is greater than 32 (over 80% of the total score), this company is under the best condition (DMU 1, DMU 2, and DMU 5). For instance, if the value of DMU 1’s annual pallet loss rate is missing, its efficiency should be $\theta_{\text{1}}^{U}$ and the efficiency of the other DMUs should take the values in the sub-scenario 1-U (the first row, Tables 2 and 3). Finally, analysts can rank these pallet rental companies based on the efficiency obtained from the proposed multi-criteria evaluation approach.

**Table 7:** Score.

DMU	Score	The selected scenario
1	35	1-U
2	35	2-U
3	23.5	3-H
4	23.5	4-H
5	33	5-U
6	14	6-L
7	18	7-L
8	14	8-L
9	16	9-L
10	15.5	10-L
11	23	11-H
12	9.5	12-L

### 3.3 Analysis

In order to examine the validity of the proposed multi-criteria evaluation approach, the results obtained from the proposed approach and those obtained from other methods are compared. Based on the proposed interval additive integer-valued DEA models with undesirable outputs, the deletion method (the deletion DEA), the dummy entries method (the dummy entries DEA), and the mean imputation method (the mean imputation DEA) are applied to measure the twelve pallet rental companies’ efficiency in each scenario. The efficiency of DMUs obtained from the deletion DEA method (deleting the variable “annual pallet loss rate”) has been shown in Table 4. According to the dummy entries method, analysts should use large enough numbers for the pallet loss rates of DMUs with missing data because the undesirable output is expected to be minimized. Therefore, the resulting efficiency of DMUs obtained from the dummy entries DEA method should be $\theta_{k}^{L}$ (under *DMU*_*k*_’s worst condition), which has been shown in Tables 2 and 3. The efficiency of DMUs obtained from the mean imputation DEA method is shown in Tables 8 and 9.

**Table 8:** Efficiency resulting from the mean imputation DEA method (DMU 1—DMU 6).

Scenario	DMU 1	DMU 2	DMU 3	DMU 4	DMU 5	DMU 6
1-M	0.241	1.000	0.189	0.272	1.000	0.346
2-M	1.000	1.000	0.189	0.274	1.000	0.346
3-M	1.000	1.000	0.189	0.272	1.000	0.346
4-M	1.000	1.000	0.189	0.454	1.000	0.346
5-M	1.000	1.000	0.189	0.272	0.445	0.346
6-M	1.000	1.000	0.189	0.272	1.000	1.000
7-M	1.000	1.000	0.189	0.272	1.000	0.346
8-M	1.000	1.000	0.189	0.272	1.000	0.346
9-M	1.000	1.000	0.189	0.272	1.000	0.346
10-M	1.000	1.000	0.189	0.272	1.000	0.346
11-M	1.000	1.000	0.189	0.272	1.000	0.346
12-M	1.000	1.000	0.189	0.272	1.000	0.334

**Table 9:** Efficiency resulting from the mean imputation DEA method (DMU 7—DMU 12).

Scenario	DMU 7	DMU 8	DMU 9	DMU 10	DMU 11	DMU 12
1-M	0.520	0.240	0.218	1.000	1.000	1.000
2-M	0.520	0.240	0.218	1.000	1.000	1.000
3-M	0.520	0.240	0.218	1.000	1.000	1.000
4-M	0.520	0.240	0.218	1.000	1.000	1.000
5-M	0.557	0.240	0.218	1.000	1.000	1.000
6-M	0.520	0.240	0.218	1.000	1.000	1.000
7-M	1.000	0.240	0.218	1.000	1.000	1.000
8-M	0.520	0.255	0.218	1.000	1.000	1.000
9-M	0.520	0.240	0.224	1.000	1.000	1.000
10-M	0.441	0.240	0.218	1.000	1.000	1.000
11-M	0.520	0.240	0.218	1.000	1.000	1.000
12-M	0.446	0.240	0.218	1.000	1.000	1.000

Then, the error rates of the four methods, i.e., the multi-criteria evaluation approach (MEA), the mean imputation DEA method (MIM), the deletion DEA method (DM), and the dummy entries DEA method (DEM), can be calculated using the formulas proposed in Subsection 3.1. The results are shown in Table 10. The average error rate of the proposed multi-criteria evaluation approach is the lowest (0.0460), while the average error rate of the mean imputation DEA method is the greatest (0.4405). The average error rate of the deletion DEA method is 0.2551, and the average error rate of the dummy entries DEA method is 0.3504. Therefore, the proposed multi-criteria evaluation approach (“interval additive integer-valued DEA models with undesirable outputs”, the “Halo + Hot deck” imputation method, and the multi-index comprehensive evaluation method) is better than the other methods, and it can help analysts measure the efficiency of DMUs with missing data.

**Table 10:** Error rate.

Scenario	MEA	MIM	DM	DEM
1	0.0000	0.7586	0.8428	0.7693
2	0.0000	0.0049	0.0000	0.0070
3	0.1076	0.0000	0.1039	0.1284
4	0.0315	0.6672	0.1043	0.0562
5	0.0201	0.6252	0.5992	0.6616
6	0.0000	1.8863	0.3088	0.0000
7	0.1382	0.9227	0.3575	0.1382
8	0.0000	0.0643	0.3407	0.0000
9	0.0175	0.0274	0.4046	0.0175
10	0.0000	0.1522	0.0000	0.0000
11	0.2365	0.0000	0.0000	2.4265
12	0.0000	0.1768	0.0000	0.0000
Average error	0.0460	0.4405	0.2551	0.3504

## 4. Conclusions

DEA, especially non-radial DEA, is a useful nonparametric technique to measure efficiency. DEA is a “data oriented” method so that analysts need to collect enough data. However, missing data is a common problem in data analysis. Therefore, it is necessary to develop effective methods to conduct DEA with missing data.

The contributions of this paper are as follows. (1) Interval additive integer-valued DEA models with undesirable outputs are proposed, which enables analysts to handle integer-valued variables and undesirable outputs when measuring efficiency. (2) The “Halo + Hot deck” imputation method is presented to deal with missing data, which is simple and easy. (3) A multi-criteria evaluation approach is proposed to measure the efficiency of DMUs with missing data based on the “interval additive integer-valued DEA models with undesirable outputs”, the “Halo + Hot deck” imputation method, and the multi-index comprehensive evaluation method. The proposed approach is applied to the pallet rental industry, and the case study proves that the proposed approach is more effective than traditional approaches such as the mean imputation DEA method, the deletion DEA method, and the dummy entries DEA method.

However, the paper still has some limitations. For example, (1) there are some other methods to deal with missing data, and the multiple imputation has been regarded as a more accurate and less biased method. It is worth combining the multiple imputation method and DEA to evaluate the efficiency of DMUs with missing data; (2) in the case study, only two inputs, one desirable output, and one undesirable output were selected because there are very few public data about the pallet rental industry. In the future, more data should be collected and the performance of pallet rental companies should be measured in more detail.

## Supporting information

S1 Code I-addIDEA-U. (DOCX)
