# Supplementary material for: Additive integer-valued data envelopment analysis with missing data: A multi-criteria evaluation approach
Source: PLoS One. 2020 Jun 11;15(6):e0234247. doi: 10.1371/journal.pone.0234247 (PMC7289371; doi:10.1371/journal.pone.0234247)
Supplement: S1 Code — (DOCX) [file pone.0234247.s001.docx]

**S1 Code. I-addIDEA-U**

MODEL:

SETS:

DMU/1..12/: p,d,k; !DMUs;

II/1..2/:o; !Inputs;

OI/1/:v; !Outputs;

FI/1/:l; !Undesirable outputs;

IV(II, DMU):x,w; !Input;

OV(OI, DMU):y; !Outputs;

FV(FI, DMU):z; !Undesirable outputs;

ENDSETS

DATA:

k=1 1 1 1 1 1 1 1 1 1 1 1;

p=0 0 0 0 0 0 0 0 0 1 0 0;

x=239 7500 310 875 284 130 101 175 144 16 109 20

10000000 460000000 92000000 40000000 9600000 3000000 3000000 10000000 8000000 22500 7000000 5000;

y=85.34 4048.3 248.6 370.72 226.03 45.21 58.07 75 49.9 1.97 313.8 1.5;

z=2 2 8 10 1 12 10 12 10 12 8 12;

ENDDATA

max=(1/4)*(@sum(FI(i):l/@sum(DMU(j):p(j)*z(i,j)))+@sum(II(i):o/@sum(DMU(j):p(j)*x(i,j)))+@sum(OI(i):v/@sum(DMU(j):p(j)*y(i,j))));

@for(II(i):

@sum(DMU(j):k(j)*d(j)*x(i,j))<=@sum(DMU(j):p(j)*w(i,j)));!Inputs;

@for(II(i):

@sum(DMU(j):p(j)*x(i,j))-o(i)=@sum(DMU(j):p(j)*w(i,j)));! Inputs;

@for(OI(i):

@sum(DMU(j):k(j)*d(j)*y(i,j))-v(i)=@sum(DMU(j):p(j)*y(i,j)));!Outputs;

@for(FI(i):

@sum(DMU(j):k(j)*d(j)*z(i,j))+l(i)=@sum(DMU(j):p(j)*z(i,j)));!Undesirable outputs;

@for(DMU(j):d(j)>=0);

@for(IV(i,j):@gin(w(i,j)));

@sum(DMU(j):d(j))=1;

end
